# The Potential of *Pontederia crassipes* to Remediate Heavy Metals in Water

**DOI:** 10.3390/plants14233604

**Published:** 2025-11-26

**Authors:** Yongming Fan, Shilong Zhang, Xiaohua Wang, Lulu Yang, Haiying Li, Kang Gao

**Affiliations:** 1 School of Human Settlements, North China University of Water Resources and Electric Power, Zhengzhou 450046, China; fanyongming@ncwu.edu.cn (Y.F.); zhangshilong0812@163.com (S.Z.); yanglulu20020126@163.com (L.Y.); 2 School of Electronic Engineering, North China University of Water Resources and Electric Power, Zhengzhou 450046, China; wangxiaohua@ncwu.edu.cn; 3 Institute of Grassland, Flowers and Ecology, Beijing Academy of Agriculture and Forestry Sciences, Beijing 100097, China

**Keywords:** heavy metal-contaminated water, remediation potential, *Pontederia crassipes*

## Abstract

Heavy metal contamination in water is a critical global environmental challenge. *Pontederia crassipes* has significant potential in phytoremediation due to its rapid proliferation and high adsorption capacity, and this review aims to synthesize its efficacy and mechanisms in removing heavy metals from water. Bibliometric analysis showed a significant increase in relevant research since 2000, with India and China as major contributors. *P. crassipes* exhibits high removal efficiencies for Cu (up to 97%), Cr (up to 85.7%), Pb (71.21–85.95%), and Zn (76.0–90.1%), along with 50–79.5% in multi-metal systems. Its remediation mechanisms involve root-dominated synergistic physical (e.g., electrostatic attraction) and chemical (e.g., ion exchange) processes. It has advantages like pH tolerance (3.5–11.0) and low cost, but faces risks of ecological invasion and secondary pollution from biomass, while its derived biochar has a stronger adsorption capacity. *P. crassipes* is an efficient phytoremediator, but rigorous management strategies are needed to mitigate risks. Future research should focus on improving efficiency and controlling invasion to preserve the ecosystem’s natural biodiversity.

## 1. Introduction

Global heavy metal contamination of water caused by industrial wastewater discharge has emerged as a critical environmental challenge. According to the United Nations Environment Programme annual report in 2021, global industrial wastewater discharge has exceeded the threshold of 40 billion metric tons per year [1], with biotoxic elements such as copper (Cu), chromium (Cr), lead (Pb), and zinc (Zn) exceeding the World Health Organization regulatory limits by over 25-fold in 32% of monitored rivers [2,3]. Current methods of physical or chemical remediation such as chemical precipitation and ion exchange (excluding phytoremediation) achieve 85–95% removal of heavy metals but face dual constraints of high unit treatment costs (50–120 USD/m^3^) and secondary pollution risks from heavy metal-containing sludge generated at 15–30% rates during treatment [4]. In this context, phytoremediation-based ecological engineering strategies have gained prominence in environmental engineering research due to their 60–80% cost reduction, eco-friendliness, and sustainability [5]. The screening and utilization of plant-based hyperaccumulators offer effective solutions for heavy metal contamination. These plants enable the in situ remediation of polluted water or soil through root/cell wall-mediated uptake, translocation, and accumulation of heavy metals, mosses (*Taxiphyllum barbieri*, *Leptodictyum riaprium* and *Vesicularia montagnei*) are used in aquatic remediation [6], *Ditrichia viscosa* for soil heavy metal immobilization [7,8], *Vetiveria zizaniodes* for both soil and water remediation [9], *Canna indica* widely applied in constructed wetlands [10], *Salvinia auriculata* for water remediation [11], *Tocoyena brasiliensis* for tropical soil remediation [12], Sudan grass for agricultural soil heavy metal removal [13], *Pistia stratiotes* and *Lemna minor* for water remediation. Notably, heavy metals exhibit distinct trophic-level toxicity, with their ionic forms and complexation directly regulating bioavailability and harm. Cr^6+^ primarily exists as Cr_2_O_7_^2−^ and CrO_4_^2−^, readily forming precipitable phosphate complexes, its half-maximal effective concentration (EC_50_) values are 2.65 mg/L for luminescent bacteria, 5.84 mg/L for activated sludge (heterotrophic respiration), 2.30 mg/L half-lethal concentration (LC_50_) for *Oryzias latipes*, 3.20 mg/L for *Daphnia magna*, and 0.30 mg/L for *Pseudokirchneriella subcapitata* (Table 1). Cu^2+^ binds to dehydrogenase active center metal sites, exerting high toxicity to invertebrates/algae (EC_50_: 0.05 mg/L for *Daphnia magna*, 0.03 mg/L for *P. subcapitata*) and moderate toxicity to microorganisms (21.4 mg/L EC_50_ for *Pseudomonas putida*) (Table 1). Zn^2+^, from ZnSO_4_·7H_2_O forms complexes with bovine serum albumin, with EC_50_ ranging from 20.93 to 22.74 mg/L for *Vibrio fischeri* (30–60 min incubation) to 0.50 mg/L for alga *Desmodesmus subspicatus*. Such microbial toxicity data often align with plant responses, reflecting shared heavy metal stress sensitivity mechanisms. Cd^2+^ and Ni^2+^ also exhibit tiered inter-organism toxicity, emphasizing the urgency of targeted remediation (Table 1). Notably, *Pontederia crassipes* (formerly *Eichhornia crassipes*) is a research hotspot due to its rapid growth and strong Cr, Cu, Pb, and Cd accumulation capacity [14,15]. Artificially constructed wetlands were able to efficiently remove Cu, Cr, Pb, and Zn via synergistic mechanisms involving uptake by *P. crassipes* plants, microbial degradation, and substrate adsorption [16].

*P. crassipes* is a floating aquatic macrophyte of the Pontederiaceae, with fibrous roots, oval leaves, and purple flowers. The purple-rooted variant, *P. crassipes* var. *purpurea*, is distinguished by its purple-colored roots. *P. crassipes* is native to the Amazon River basin in South America. Since the 19th century, it has been introduced to various regions worldwide for purposes such as water purification and ornamental use, including Asia (China, India, Bangladesh), Africa (Egypt, South Africa, Ethiopia), North America (the United States, Mexico), and Oceania (Australia). However, due to its strong reproductive capacity, it has become an invasive species in many introduced regions, causing ecological problems [14].

This review systematically summarizes the remediation potential of *P. crassipes* in heavy metal-contaminated water, analyzing its overall effectiveness in removing heavy metals from water, core mechanisms, ecological adaptability, resource utilization value, and potential application risks. It provides theoretical support for the rational application of *P. crassipes* in water remediation and offers guidance for the formulation of future research directions. The review highlights the ability of *P. crassipes* to effectively remove metals such as Cu, Cr, Pb, and Zn through rapid growth and adsorption mechanisms, including physical adsorption, ion exchange, and complexation, while demonstrating dynamic synergistic regulation in multi-metal contamination scenarios. *P. crassipes* exhibits broad ecological adaptability, low maintenance costs, and high resource utilization value.

## 2. Research Progress and Current Status

### 2.1. Bibliometric Data Integration Strategy and Research Progress

A comprehensive literature search was conducted across two major academic databases to investigate research output related to *P*. *crassipes*/*E*. *crassipes* and heavy metals. In CNKI database, searches using the Boolean operator *Eichhornia crassipes* and heavy metals in the title, abstract, and keyword fields retrieved 264 documents (including both dissertations and journal articles) as of 7 May 2025. Conversely, identical searches with the taxonomic synonym *P. crassipes* yielded only one relevant document. Parallel searches in the WoS demonstrated similar trends, with *Eichhornia crassipes* and heavy metals returning 360 publications, compared to merely 7 records for *P. crassipes* and heavy metals using the same search parameters and cutoff date. The language filter for CNKI is set to Chinese and foreign languages, while WoS has an all-languages option. After removing duplicates and screening the title and abstract to exclude studies unrelated to *E. crassipes*, *P. crassipes*, or its heavy metal removal capabilities, 632 articles were retained for analysis (data until 7 May 2025). Bibliometric analyses were performed using VOSviewer (version 1.6.20, CWTS), (Leiden University, The Netherlands) for keyword co-occurrence mapping and Scimago Graphica (version 1.0.46, SCImago Research, Madrid, Spain) for visualizing international collaborations. Data integration and visualization were supported by Adobe Photoshop 2020 (version 21.0.037, Adobe Inc., San Jose, CA, USA) and WPS Office (version 12.1.0.18276, Zhuhai Kingsoft Office Software Co., Ltd., Zhuhai, China). This workflow allowed us to clarify the current research landscape of *P. crassipes* in heavy metal remediation while revealing emerging trends and hotspots.

The remediation potential of *P. crassipes* in heavy metal-contaminated water has been widely studied. Dai and Che systematically assessed its purification efficiency for Pb, Cd, and Cr, demonstrating distinct performance in composite contamination systems such as industrial wastewater, agricultural runoff, and mining effluents [18]. The removal efficiency of Pb was highest (71.21–85.95%), followed by Cd (61.54–83.33%), and Cr (29.52–58.62%). The core period for measuring the purification effect of *P. Crassipes* on heavy metals in the study is six days, and all key data on purification load and purification efficiency are derived based on this period. Their study identified pH and initial metal concentration as critical factors influencing remediation efficacy, establishing a foundation for subsequent applications, and found that the ability of water hyacinth to purify mercury (Hg) was most effective at neutral pH with uptake efficiency decreasing as initial Hg concentration increased, its adsorption capacity for Pb was inversely proportional to initial Pb concentration, and its adsorption of Cd was relatively lower but still effective, with heavy metals primarily concentrated in the roots [19]. Mishra and Tripathi elucidated Cr and Cu remediation mechanisms, demonstrating root-mediated metal immobilization through adsorption and ion exchange combined with leaf bioaccumulation to reduce their aqueous concentrations, with maximum removal efficiencies of 95% and 91% for Cu by *E. crassipes* and *Pistia stratiotes* at 2 mg/L, and 85% and 83% for Cr by *E. crassipes* and *Spirodela polyrrhiza* at 1 mg/L [14]. Lu et al. reported removal efficiencies of 70% for Zn, 65% for nickel (Ni), and 50% for arsenic (As) in multi-metal systems, for example, Zn removal efficiency reached 70% under specific conditions, Ni was removed at a rate of 65% in similar scenarios, and As removal efficiency was approximately 50% in the experimental setups, emphasizing pH and temperature as key regulatory parameters that can influence these removal efficiencies [19]. Rezania et al. validated the Hg removal capacity of *P. crassipes* with an accumulation of 1.99 mg/g dry weight in the root tissues and a Cd removal capacity of nearly 100% from an aqueous solution within 1 h at an initial Cd concentration of 50 mg/L, and highlighted practical applications in industrial wastewater treatment [20].

### 2.2. Bibliometric Analysis and Publication Statistics

**Figure 1:**
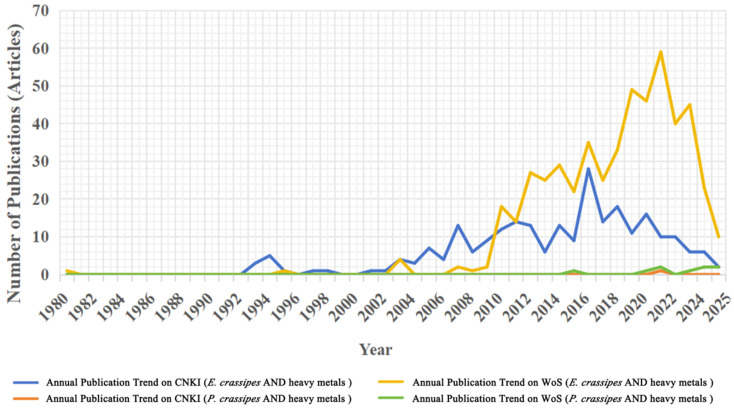
The literature on *P. crassipes*/*E. crassipes* and heavy metals, retrieved from CNKI and WoS, during 1980–2025 (as of 7 May 2025). Due to few publications on *P. crassipes* and heavy metals (1 in CNKI, 9 in WoS), the dataset is insufficient for statistically meaningful analysis. Articles include scholarly papers (peer-reviewed journal publications) and academic dissertations/theses. Retracted articles were excluded from the bibliometric analysis.

Analysis of annual publication volumes from the China National Knowledge Infrastructure (CNKI) and Web of Science Core Collection (WoS) reveals a significant increase in research output related to *P. crassipes* and heavy metals after 2000 (Figure 1). This trend aligns with heightened global attention to environmental challenges, specifically water pollution and phytoremediation technologies. From 1980 to 2000, studies on *P. crassipes* and heavy metals were scarce (Figure 1), a pattern attributable to the socioeconomic priorities of that era. During the early stages of economic globalization and the internet and information age, global agendas prioritized economic development over environmental protection, leading to limited societal engagement with ecological issues and constrained research progress [21]. Between 2000 and 2015, publications on *P. crassipes* and heavy metals showed substantial growth (Figure 1). This shift correlates with the establishment of the United Nations Sustainable Development Goals in 2000, which environmental concerns reaching global prominence [22]. Increased international collaboration on climate change mitigation, pollution reduction, and biodiversity conservation during this period reflects heightened societal awareness of ecological challenges, thereby stimulating expanded research efforts. Since 2015, research output has continued to rise, peaking notably in 2022, indicating sustained scientific interest in ecological restoration and accelerated advancements in related fields.

CNKI and WoS publication trends demonstrated similar growth trajectories after 2010. Post-2020, WoS output declined, whereas CNKI publications experienced a temporary decrease in 2021 before rebounding in 2022–2023. This resurgence likely reflects growing global interest in bioremediation technologies, particularly the use of *P. crassipes* and other plants for treating heavy metal-polluted water. The post-2020 decline may also stem from disruptions to research activities caused by the COVID-19 pandemic. Publication volumes confirm that *P. crassipes* remains a prominent subject in research related to aquatic heavy metal removal, with sustained academic interest persisting, despite the post-2020 fluctuations (Figure 1).

**Figure 2:**
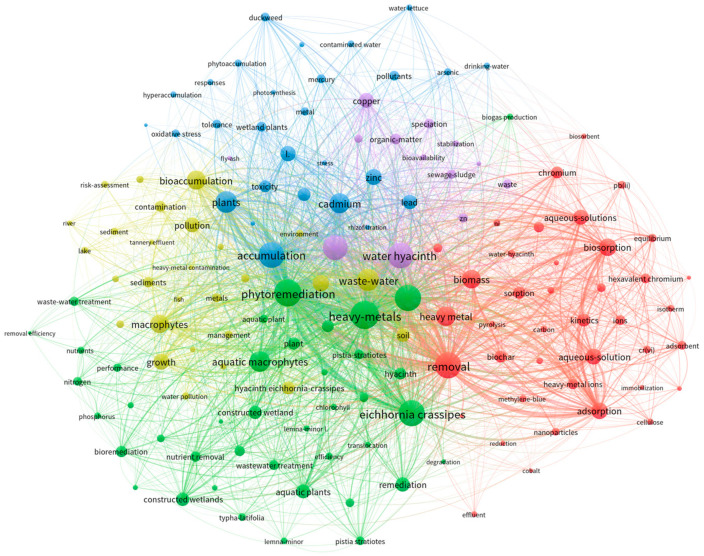
Research hotspot analysis. a, Keywords with occurrence frequency >5 since 2020. Each node represents a keyword (size proportional to frequency, larger nodes = core research themes), and links denote co-occurrence (thickness proportional to frequency). Color-coded modules categorize heavy metal pollution remediation technologies (each color = an algorithmic cluster): Yellow (ecological restoration, including plant-based water purification and wetland systems). Green (specific aquatic plants in wetland treatment). Blue (bioaccumulation and plant responses to heavy metals). Purple (heavy metal pollutants), Red (physicochemical remediation methods). Due to insufficient literature on *P. crassipes* and heavy metals (1 in CNKI, 9 in WoS), keyword co-occurrence analysis for this term was excluded (methodological requirements unmet). This figure was created with VOSviewer (version 1.6.20.0, Centre for Science and Technology Studies, CWTS, Leiden University, The Netherlands). Cut-off date: 7 May 2025.

Figure 2 maps the *P. crassipes* node and analyzes its keyword associations. The node displays a strong co-occurrence with heavy metals and removal, underscoring its remediation potential in contaminated water as a major research focus. Highly central keywords such as adsorption and biosorption highlight the emphasis on adsorption mechanisms and biological accumulation. The co-occurrence network clarifies research hotspots and trends in *P. crassipes*-mediated heavy metal remediation. Figure 2 shows that the accumulation and removal of heavy metals in water is the focus of this research, and that this plant is widely regarded as an important phytoremediation tool for polluted aquatic systems. However, *P. stratiotes* appears twice in the figure, in the green and red areas. This repeated keyword may be due to its representativeness in related studies or its mention in different research contexts. *P*. *stratiotes* and *Lemna minor* are keywords because of their unique biological properties. They are commonly used in water remediation studies, especially in research on multiple plants in phytoremediation. As common aquatic plants, they appear in many papers on this topic.

### 2.3. International Collaboration and Linkages

The global research on *P. crassipes*-mediated heavy metal remediation is mainly concentrated in Asia (China, India), Africa (Egypt, Pakistan), and South America (the native region). Among them, China and India are the major contributors, accounting for more than 50% of the total publications (Table 2). India leads globally with 100 publications, supported by 2941 total citations and 29.41 citations per paper, demonstrating both productivity and impact. China follows with 68 publications and 2071 total citations, achieving 30.46 citations per paper. Despite its smaller output, China maintains research influence comparable to that of India. Egypt has significant regional impact in Africa and the Middle East with 23 publications, 792 total citations, and 34.43 citations per paper. Pakistan ranks fourth with 19 publications and 545 total citations, though its lower citation rate (26.45 per paper) suggests prioritization of output quantity over immediate scholarly impact. Saudi Arabia (total publication count: 19) ranks fifth with the lowest citation rate (26.31 per paper), indicating limited global influence of its research outcomes.

Institutional contributions highlight the prominence of research institutions in Saudi Arabia and Egypt (Table 1). King Khalid University in Saudi Arabia leads with 6 publications and 133 total citations, achieving 22.17 citations per paper. This performance significantly surpasses the national average of 12.29 citations, showcasing the institution’s specialized advancements in technological applications. Egypt’s National Institute of Oceanography and Fisheries produced 6 publications, accumulating 113 total citations with 18.83 citations per paper, highlighting its expertise in marine and freshwater ecosystem restoration. Notably, Tanta University in Egypt distinguishes itself with 4 publications, attaining the highest global citation average (38.00) and 152 total citations, indicating a focus on high-impact innovations or critical regional environmental challenges. Government College University in Pakistan ranks among top contributors with 4 publications and 25.00 citations per paper, reflecting South Asia’s potential in advancing low-cost water remediation technologies.

This analysis reveals India and China as global leaders in *P. crassipes*-mediated heavy metal remediation research, while Egypt and Saudi Arabia leverage institutional specialization to address regional priorities. The high output but moderate impact of Saudi Arabian institutions suggests the need for enhanced international collaboration, whereas Egypt’s quality-over-quantity model offers lessons for resource-constrained regions.

**Figure 3:**
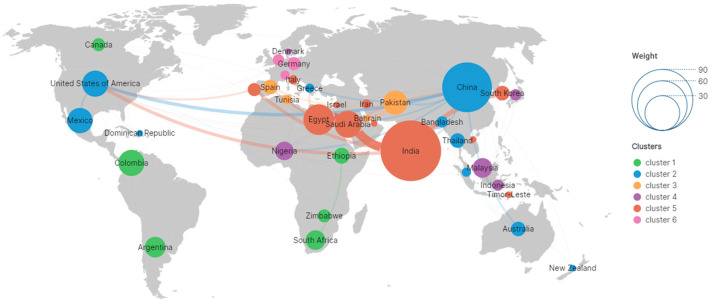
A global academic publication co-authorship network map. This map analyzes the distribution of core research countries, features of international collaboration networks, and potential research directions/challenges, with visual cues: node size = publication volume, color = collaborative relationships, line thickness = collaboration intensity. Color ranges visually show the general communication scope between regions. Created with Scimago Graphica (Scimago Lab, Madrid, Spain, version 2.3.1). Cut-off date: 7 May 2025.

The research network map (Figure 3) was constructed based on the keywords *P. crassipes* and heavy metals. Global research on heavy metal remediation by using water hyacinth has been predominantly led by developing countries. European countries like France, Germany, Italy, and Spain are also involved in this research field. In terms of collaborative networks, partnerships such as China-South Africa and India-Nigeria are noticeable, showcasing national scientific and technological cooperation, while Southeast Asian research entities are actively integrating into the global network through international collaborations.

## 3. Remediation Efficacy of Heavy Metals by *P*. *crassipes*

### 3.1. Copper (Cu)

*P. crassipes* exhibits species-specific efficacy in Cu^2+^ remediation, initiated by rapid and efficient adsorption processes, which is tightly linked to its unique root structure, cell wall composition, and physiological mechanisms, distinguishing it from other aquatic macrophytes. The removal Cu^2+^ involves a synergistic combination of physical and chemical adsorption mechanisms, both finely tuned to the plant’s intrinsic biological properties.

In the physical adsorption process, *P. crassipes* passively intercepts Cu^2+^ mainly through its specialized root system. Unlike the fibrous roots of many aquatic plants, *P. crassipes* develops a dense, branched fibrous root network with abundant root hairs and micropores (2–5 μm in diameter) on the root surface. These micropores, along with the porous architecture of the root cell walls-rich in cellulose, hemicellulose, and pectin—create a physical trap that efficiently sequesters Cu^2+^ ions from water. This physical adsorption conforms to the Freundlich isotherm model [23], reflecting the heterogeneity of adsorption sites on the roots. The core driving force of this process is the electrostatic attraction between negatively charged functional groups (primarily carboxyl groups, –COO^−^) on the root surface and positively charged Cu^2+^ ions [23,24] which adheres to Coulomb’s law [25]. Notably, the carboxyl groups in *P. crassipes* roots are predominantly distributed in the pectin layer of the root epidermis and root hairs-anatomical features that maximize the exposure of negative charge sites, enhancing Cu^2+^ capture compared to other aquatic plants with fewer root hairs.

The adsorption efficiency of *P. crassipes* for Cu^2+^ is highly dependent on pH, and this dependence is closely tied to the plant’s own physiological adaptability. Within the optimal pH range of 3.5–5.5, carboxyl groups on *P. crassipes* roots remain fully deprotonated (maintaining –COO^−^ form), maximizing the number of available cation adsorption sites [26,27]. More importantly, *P. crassipes* exhibits stronger root vitality and metabolic activity under acidic conditions (pH 3.5–5.5): studies have shown that its root relative growth rate under pH 4.0 is 6.12-fold higher than that in alkaline environments (pH 11.0) [25], which further promotes the development of root micropores and the expression of surface functional groups, indirectly enhancing physical adsorption. When pH drops below 3.3, protonation reverses the surface potential of *P. crassipes* root cells to positive, intensifying electrostatic repulsion with Cu^2+^, at the same time, low pH inhibits the synthesis of root cell wall pectin (a key source of carboxyl groups), leading to a drastic reduction in Cu^2+^ removal efficiency [28].

Chemical adsorption of Cu^2+^ by *P. crassipes* is mediated by the species-specific synergistic effects of ion exchange, complexation, and electron donor–acceptor interactions [28]. Owing to the plant’s unique root exudates and cell wall composition, its adsorption mechanisms are distinctly different from those of general aquatic plants. During ion exchange, Ca^2+^ released from the pectin matrix of P. crassipes root cell walls specifically displaces Cu^2+^ in the aqueous phase [24]. In contrast to other plants that release a mixture of K^+^, Na^+^, and Ca^2+^, *P. crassipes* predominantly releases Ca^2+^, a feature attributed to the high calcium pectate content in its root cell walls (~35% of dry weight). Concurrently, functional groups unique to *P. crassipes* roots, including carboxyl groups (from pectin), amino groups (from root surface proteins), and hydroxyl groups (from cellulose)—form stable five- or six-membered ring complexes with Cu^2+^ through coordination bonds [23]. Specifically, the amino acid sequence of *P. crassipes* root surface proteins contains a high proportion of aspartic acid and glutamic acid (rich in carboxyl side chains), which act as chelating centers for Cu^2+^, enhancing complex stability. Electron donor–acceptor interactions further improve adsorption efficiency: the –CH groups in *P. crassipes* root cell wall lignin serve as electron donors, transferring electrons to Cu^2+^ (acting as an electron acceptor) to form stable surface complexes, a process that is not observed in aquatic plants with low lignin content [29].

Similarly to physical adsorption, chemical adsorption efficiency of *P. crassipes* for Cu^2+^ is regulated by pH, with peak Cu^2+^ removal (75%) achieved at pH 4.0–6.5 through the combined action of ion exchange and complexation (Table 3) [28]. At this pH range, not only are carboxyl groups fully deprotonated to facilitate ion exchange, but *P. crassipes* also secretes more low-molecular-weight organic acids from its roots, these acids can act as auxiliary ligands to enhance the complexation of Cu^2+^ with root surface functional groups. When pH exceeds 6.5, Cu^2+^ in the solution tends to hydrolyze into Cu(OH)_2_ precipitates, which are less likely to be complexed by root functional groups; meanwhile, high pH inhibits the secretion of organic acids by *P. crassipes* roots, weakening chemical adsorption.

**Figure 4:**
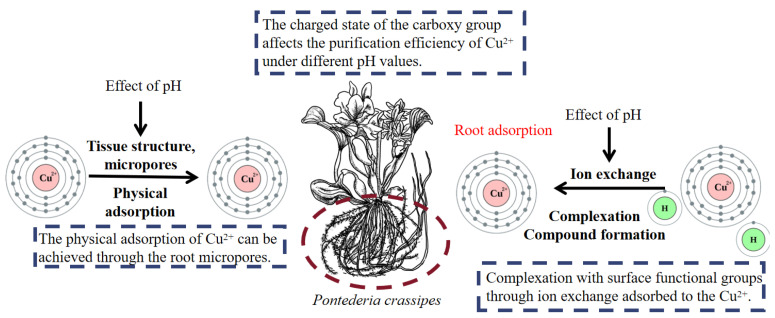
The mechanism of Cu^2+^ removal by *P. crassipes*. Complexation refers to the formation of a coordination complex between a metal ion (Cu^2+^) and molecules/functional groups that act as ligands (carboxyl, amino, hydroxyl groups). The botanical illustration of *P. crassipes* was created using Procreate (version 5.3.15).

The cooperative interplay of physical and chemical adsorption (Figure 4) underpins the rapid and high efficacy of *P. crassipes* in adsorbing Cu from contaminated water. The strong complexation and ion exchange ensure that adsorbed Cu is stably retained in the root biomass, minimizing the risk of desorption. Compared to other common aquatic phytoremediators (*Spirodela polyrrhiza* with ~60% Cu removal efficiency), *P. crassipes* achieves a maximum Cu removal efficiency of 97% (Table 3), which is largely due to its specialized root structure, unique cell wall composition, and pH-adapted physiological mechanisms—all of which are species-specific traits that make it a superior candidate for Cu-contaminated water remediation [26].

### 3.2. Chromium (Cr)

**Figure 5:**
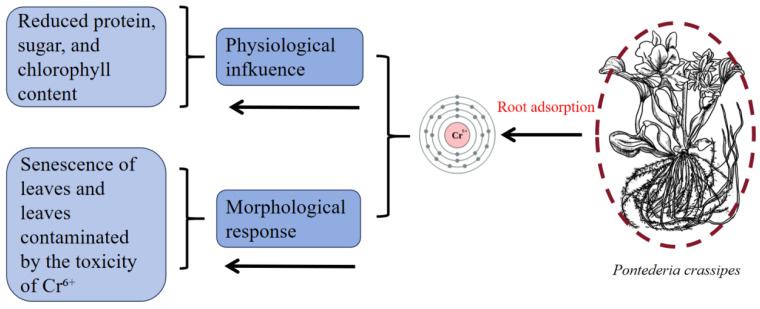
The effect of Cr^6+^ on *P. crassipes*. The botanical illustration of *P. crassipes* was created using Procreate (version 5.3.15).

Cr predominantly exists in the oxidation states of Cr^3+^ (trivalent form) and Cr^6+^ (hexavalent form) within environmental systems [30]. These Cr species have marked disparities in their physicochemical properties and toxicological profiles, with Cr^6+^ constituting a priority environmental contaminant given its elevated toxicity potential [31]. Cr^6+^ exhibits dual environmental threats through human carcinogenicity and ecosystem disruption mechanisms mediated by its potent oxidative capacity and membrane permeability [32]. Elevated exposure to Cr^6+^ reduces protein synthesis, carbohydrate metabolism, and chlorophyll production in *P. crassipes*, consequently disrupting core physiological functions that manifest as characteristic phytotoxicity symptoms, including chlorotic tissue development and biomass suppression [33]. To mitigate metal stress (Cr^6+^), *P. crassipes* employs an adaptive detoxification strategy through regulated leaf abscission mechanisms that selectively excise metal-laden tissues [34]. This physiological adaptation effectively minimizes Cr bioavailability while preserving vital metabolic processes (Figure 5).

Cr bioremediation strategies for aquatic systems primarily utilize rhizofiltration by whole *P. crassipes* plants coupled with biochar, which is produced from entire *P. crassipes* plants [35,36]. Throughout tested concentration ranges, Cr^6+^ solutions exhibited no significant phytotoxicity in *P. crassipes*, and organ-specific partitioning analysis demonstrated significantly enhanced Cr^6+^ retention capacity in root tissues relative to stems and leaves, with removal rates persisting across concentrations (Table 4). The phytoremediation efficiency of *P. crassipes* was inversely correlated with aqueous Cr^6+^ concentration, showing decreasing removal rates from 84% at 1.0 mg/L to 63% at 20.0 mg/L [37,38]. Atomic adsorption spectrophotometric quantification verified 84% Cr^6+^ removal efficacy, consistent with the plant’s bioaccumulation ability, demonstrating predominant heavy metal sequestration in root systems, followed by leaves and stems [38].

**Figure 6:**
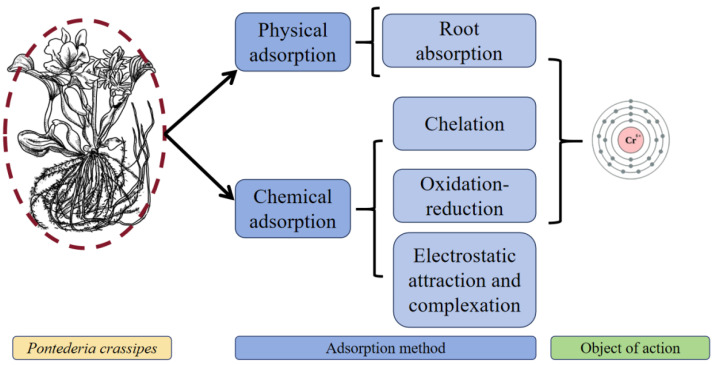
The classification of Cr^6+^ removal methods by *P. crassipes*. The botanical illustration of *P. crassipes* was created using Procreate (version 5.3.15).

The root system of *P. crassipes* removes Cr^6+^ through four coordinated processes (Figure 6). In physical adsorption, Cr^6+^ binds to functional groups on the root surface such as carboxyl and phosphate via electrostatic interactions, forming surface complexes. During chemical adsorption and chelation, root-secreted phytochelatins stabilize Cr^6+^ through hexadentate coordination. Redox reactions catalyzed by root-specific chromate reductases reduce Cr^6+^ to less toxic Cr^3+^, and this is accompanied by activation of the electron transfer chain. Transmembrane compartmentalization involves NRAMP transporter-mediated Cr^3+^ transport into vacuoles via proton gradient-driven vesicular transport, enabling metal redistribution through xylem loading. These processes synergistically form a multi-barrier system that transforms Cr^6+^, mitigates toxicity, and regulates inter-tissue translocation [38].

The mechanism of Cr immobilization in *P. crassipes* operates through dual-mode adsorption processes comprising distinct physical and chemical pathways (Table 5), governed by the plant’s structural organization. Physical adsorption mechanisms involve three synergistic components: (1) electrostatic interactions between root cell wall polysaccharides, proteins, and HCrO4− ions under acidic conditions mediated by Coulombic forces [38]. (2) concentration-gradient-driven diffusion trapping in porous matrices of stems and leaves [37]. (3) secondary physical adsorption through weak interfacial forces (hydrogen bonding and van der Waals interactions) at root surface –CH groups [39]. Chemical adsorption processes dominate as 1) redox transformation where –CH groups oxidize to carboxyl moieties (–COO-, –C=O) while reducing Cr^6+^ to Cr^3+^ [38], and 2) ligand-specific coordination via amino, carboxyl groups from intracellular metabolites chelating Cr^6+^ [39]. A unique chemisorption pathway involves γ-Fe_2_O_3_- mediated hydroxylation, where protonated -OH^2+^ sites chemically immobilize CrO_4_^2−^ through covalent bonding [39]. These adsorption pathways are predominantly pH-dependent, with physical adsorption efficiency controlled by surface area and pore architecture, whereas chemical adsorption facilitates the transformation of Cr^6+^ via electron transfer or coordination chemistry.

### 3.3. Lead (Pb)

**Figure 7:**
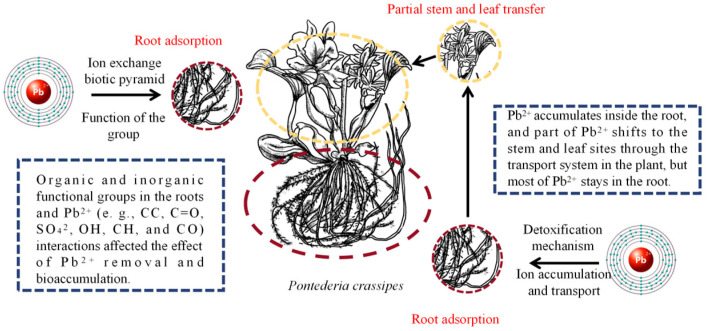
The mechanism of Pb^2+^ removal by *P. crassipes*. The botanical illustration of *P. crassipes* was created using Procreate (version 5.3.15). Ion exchange biotic pyramid describes the mechanism by which Pb^2+^ enters the plant through root ion exchange and subsequently undergoes a pyramid-like hierarchical process of translocation and retention within the organism. Specifically, it includes: (1) Base layer (Adsorption): Functional groups on the root surface capture Pb^2+^ via ion exchange. (2) Intermediate layer (Uptake and Translocation): Pb^2+^ crosses membranes into cells, with partial translocation to aerial parts via the vascular system. (3) Apex layer (Retention and Detoxification): Most Pb^2+^ is immobilized in the roots, forming the biological enrichment apex of the pyramid.

The root system of *P. crassipes* immobilizes Pb^2+^ predominantly through a combination of physical and chemical adsorption (Figure 7), facilitated by electrostatic interactions and pore entrapment within polysaccharide-rich cell walls [40]. The hierarchical architecture of root cell walls, characterized by porous structures and biopolymer matrices (e.g., cellulose, lignin, pectin), provides abundant adsorption sites [41]. Synergistic interactions among these biopolymers enhance Pb^2+^ retention efficiency by optimizing surface charge distribution and pore accessibility [42,43]. The root cell walls of *P. crassipes* are rich in polysaccharides (cellulose, lignin, pectin) with a hierarchical porous structure, this architecture provides 2–3 times more adsorption sites for Pb^2+^ than the roots of *Vetiveria zizaniodes* [42]. The high specific surface area (12.5 m^2^/g) of *P. crassipes* roots further enhances Pb^2+^ entrapment [43]. Furthermore, the high specific surface area of the roots amplifies their adsorption capacity [42].

Chemical adsorption contributes significantly to Pb^2+^ immobilization (Figure 7). Functional groups within root cell walls, such as hydroxyl (–OH), carboxyl (–COOH), and amino (–NH2) groups, coordinate with Pb^2+^ through ion exchange and complexation. Oxygen-containing functional groups, particularly carboxyl and hydroxyl groups, preferentially form stable five-membered chelate complexes with Pb^2+^. The *P. crassipes* root system adsorbs Pb^2+^ onto cell walls through a cation exchange mechanism, while simultaneously releasing other cations. Functional groups (–OH, –COOH, –NH_2_) on *P. crassipes* roots form stable five-membered chelate complexes with Pb^2^+, for example, –COOH groups react with Pb^2^+ to form Pb-OOC-R complexes, with a binding constant of 10^5^.^2^ (measured via potentiometric titration) [44]. Crucially, over 80% of adsorbed Pb^2+^ remains stably retained in *P. crassipes* roots, with strong chemical bonding significantly reducing the potential for desorption. This root-dominant accumulation reduces Pb-induced phytotoxicity and facilitates targeted biomass harvesting for Pb removal [45].

### 3.4. Zinc (Zn)

The fibrous root system of *P. crassipes* establishes a three-dimensional network that physically intercepts insoluble particles and colloidal matter, enabling preliminary concentration of Zn^2+^ at the root–water interface [46]. This process is amplified in the purple-rooted variant (*P. crassipes* var. *purpurea*), which exhibits root biomass 7–20 times greater than common cultivars, thereby enhancing surface area for particle entrapment. Structural advantages such as high specific surface area and microporous architecture in roots and leaves provide abundant binding sites, while porous cellulose and lignin configurations further optimize adsorption efficiency. Zn^2+^ removal rates of 76.0% and 90.1% for common and purple-rooted variants, respectively, emphasize the role of physical architecture in adsorption capacity [46].

**Figure 8:**
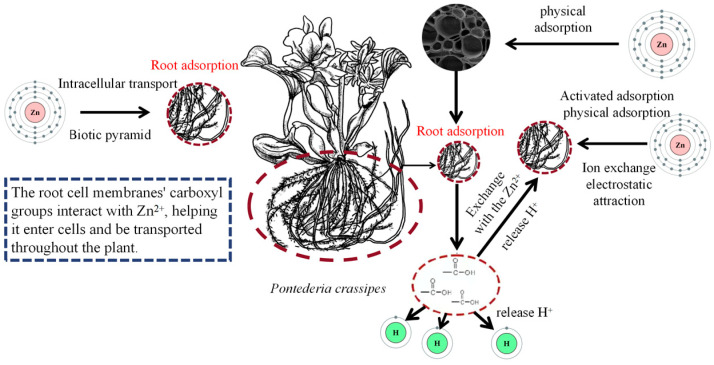
A flowchart of the mechanism of Zn^2+^ removal. The botanical illustration of *P. crassipes* was created using Procreate (version 5.3.15).

Chemical adsorption in *P. crassipes* involves ion exchange and ligand coordination mediated by functional groups on root cell walls, including carboxyl (–COOH) and hydroxyl (–OH) groups (Figure 8) [47]. During the initial phase, deprotonated carboxyl groups (–COO-) electrostatically bind Zn^2+^ via nonspecific interactions, temporarily immobilizing ions within pores of polysaccharides and proteins. In the subsequent phase, stable metal–organic complexes (e.g., Zn-OOC-R) form through ionocovalent bonds, driven by ATP-dependent transmembrane transport. Organic secretions such as humic acids and polysaccharides enhance adsorption by chelating Zn^2+^, while bioaccumulation confines adsorbed ions predominantly within root cells, minimizing re-release. In this study, the purification time of *P. crassipes* for Zn^2+^ (i.e., the contact time in the adsorption experiment) is 3 h [47]. Adsorption efficiency is pH-dependent, peaking in neutral or mildly acidic conditions where carboxyl group deprotonation is maximized. The formation of stable complexes (Zn-OOC-R) ensures that once adsorbed, Zn is not easily desorbed. The purple-rooted variant achieves superior stability (90.1% removal) compared to common strains (76.0%), attributable to enhanced functional group activity and structural synergy [46].

### 3.5. Remediation Efficacy for Multi-Metal Contamination

In practical water remediation scenarios in which there is heavy metal contamination, single-metal pollution accounts for less than 20% of cases, while over 80% involve composite systems containing two or more metals [48]. These multi-metal systems often exhibit nonlinearly amplified toxicity effects [49], posing dual challenges for remediation technologies that require simultaneous removal of multiple metals and mitigation of efficiency losses caused by ionic competition.

*P. crassipes* demonstrates dual adsorption characteristics involving synergistic and competitive interactions in composite heavy metal-polluted water. Research shows dynamic regulatory mechanisms in competitive adsorption between coexisting Cu^2+^ and Cd^2+^, where the root surface exhibits higher affinity for Cu, enabling Cu^2+^ to preferentially occupy adsorption sites and suppress Cd^2+^ uptake [50]. Conversely, interactions between Cu^2+^ and Cr^6+^ follow an inverse mechanism. Rapid Cu^2+^ binding to amino and carboxyl groups inhibits amino-to-amide transformations and prevents consumption of the carboxyl group during Cr^6+^ reduction, thereby prolonging electrostatic attraction for Cr removal. This process enhances Cr^6+^ removal efficiency by 15% without compromising the elimination of copper [28]. Synergistic effects in Pb-Zn-Cd-Mn systems achieve removal efficiencies exceeding 79.50% for Pb and Mn, with lower rates for Zn (64.21%) and Cd (50.00%), low concentrations of Pb, Cd, and Mn promote Zn removal, whereas Zn suppresses the elimination of these metals [44].

The plant’s remediation mechanism operates through a root-dominant accumulation pattern optimized for sequestration resistance. Roots consistently serve as the primary accumulation site across contamination scenarios, including Pb-Zn-Cd-Mn [44] and multi-metal systems containing Cd, Co, Cr, Cu, Fe, Mn, Ni, Pb, and Zn [51]. Bioconcentration factor (BCF) values consistently exceed 1.0 (BCF >1.0 in multi-metal systems), confirming efficient aqueous metal accumulation. Translocation factor (TF) values below 1.0 reflect a stress-resistance strategy that minimizes metal transfer to stems and leaves, reducing toxicity to physiologically active organs [51]. This root sequestration enables simultaneous treatment of nine heavy metals (Cd, Co, Cr, Cu, Fe, Mn, Ni, Pb, Zn), highlighting its potential for composite pollution remediation [51].

*P. crassipes* maintains stable performance across contamination contexts, achieving removal efficiencies of 50–79.5% in Cd-Cu [50], Cr-Cu [28], Pb-Zn-Cd-Mn [44], and multi-metal systems [51]. It exhibits superior Pb and Mn removal (>79.5%) while retaining baseline Zn remediation capacity despite inhibitory effects. This broad-spectrum tolerance establishes this plant as a superior candidate for bioremediation [28,44,50,51].

## 4. Advantages and Limitations of *P. crassipes* in Remediation of Heavy Metal-Contaminated Water

### 4.1. Advantages of P. crassipes in Heavy Metal Remediation

#### 4.1.1. Ecological Adaptability and Remediation Capacity of *P. crassipes*

The minimum water temperature required for the normal growth of *P. crassipes* is 12 °C, and it is not frost-tolerant (above-ground parts will wither when the temperature is below 0 °C). Comparative studies on aquatic plants’ ecological adaptation to heavy metal-polluted water identify distinct performance patterns among six species, including *Ipomoea aquatica*, *Brassica campestris*, *Canna indica*, *P. crassipes*, Alternanthera philoxeroides, and *P*. *stratiotes* [27]. In evaluations of composite heavy metal tolerance, *P. crassipes* ranks second in comprehensive metal tolerance index (MTI) for metals such as Hg, Cd, Pb, Cr, Cu, and Zn, outperforming all tested species except *C. indica* [27]. Its rapid growth facilitates swift establishment of dominant populations in contaminated water, enabling large-scale biomass production for efficient purification. Histological root analyses reveal dual mechanisms of heavy metal retention via surface adsorption and nutrient assimilation through transmembrane transporter-mediated active adsorption, forming a synergistic system for combined remediation of heavy metals and eutrophication [27].

The purple-rooted variant of *P. crassipes* exhibits enhanced adaptive responses under Cd and temperature stress. Low-concentration Cd exposure triggers endogenous cytoprotective mechanisms, including chelating peptide and antioxidant enzyme synthesis, to mitigate toxicity. However, high Cd concentrations inhibit growth, indicating a tolerance threshold. Combined low-temperature and Cd stress exacerbates physiological dysfunction, necessitating stringent control of Cd levels and ambient temperatures in practical applications [52]. Environmental adaptability studies confirm broad pH tolerance (pH 3.5–11.0), with optimal physiological activity in acidic conditions (pH 3.5). Root vitality and relative growth rates under acidic conditions (6.12-fold increase) significantly exceed those in alkaline environments (3.08-fold at pH 11.0), reflecting enhanced metabolic activity in acidic settings [53].

Compared to *P*. *stratiotes*, *Spirodela polyrhiza*, *Lemna minor*, and *Elodea nuttallii*, *P. crassipes* demonstrates superior heavy metal remediation efficiency. It achieves higher removal rates for diverse metals, including Cu, Cd, Cr, Pb, Zn, Mn, Ni, Hg, and As, with broader applicability and stability in contaminated systems [54]. Specifically, *P. crassipes* outperforms species like *Juncus effusus* and *Oenanthe javanica* in Zn remediation, accumulating higher Zn levels in roots, stems, and leaves while maintaining stable growth under high Zn stress [55].

The species achieves efficient multi-metal remediation by synergistically integrating three critical attributes: high accumulation capacity, robust stress tolerance, and broad environmental adaptability. While promising, scaling this approach necessitates coupling with plant-microbe systems to amplify remediation efficiency and secure long-term ecological sustainability [54,55].

#### 4.1.2. Ecosystem Service Functions and Ecological Risks of *P. crassipes*

*P. crassipes* demonstrates dual ecological characteristics marked by coexisting ecosystem service functions and invasive risks. As a bioenergy resource, the plant enhances biogas production through hydrolysis processes and can be processed into biomass pellets with a calorific value of 14.55 MJ/kg [56]. In wastewater treatment, it effectively mitigates eutrophication by absorbing nutrients such as nitrogen, phosphorus, and potassium [57], while also exhibiting potential for remediating heavy metal contamination [51]. Agriculturally, it serves as composting material, and its cellulose content enables extraction for membrane fabrication [58,59]. Invasive species present compounded ecological risks by disrupting environmental stability and socioeconomic systems through their rapid proliferation, which not only degrades water quality via diminished dissolved oxygen levels and altered nutrient concentrations, but also obstructs hydrological connectivity and navigation infrastructure through the formation of dense surface mats while simultaneously undermining the productivity of local fisheries thereby needing the development of threshold-based management frameworks that strategically synchronize biomass control measures with systematic harvesting protocols to preserve equilibrium between ecosystem optimization and risk reduction while enabling sustainable resource utilization [56].

#### 4.1.3. Enhanced Remediation Efficiency Using *P. crassipes*-Derived Biochar

*P. crassipes* demonstrates dual remediation mechanisms for heavy metal contamination, integrating phytoremediation with engineered biochar derived from thermochemical biomass conversion that exhibits synergistic enhancement effects [60]. Conventional biochar production predominantly employs plant biomass pyrolysis, where variations in feedstock pretreatment critically determine contaminant removal efficiency [61]. Magnetic *P. crassipes* biochar (MBC), synthesized through iron salt and potassium carbonate activation, demonstrates Cr removal via monolayer chemisorption mechanisms [39]. At pH 2, Cr^6+^ adsorption of MBC correlates with changes in oxyanion speciation, while solution pH regulates adsorption processes through three pathways: modification in chromium speciation, electrostatic/redox interaction adjustment, and functional group activation. Maximum Cr^6+^ removal under acidic conditions stems from reductive CH-group activity combined with γ-Fe_2_O_3_-induced electrostatic-complexation synergism. MBC exhibits magnetic separation capability enabling regeneration cycles while monocomponent *P. crassipes* biochar displays inherent alkalinity (pH 9.38), inducing solution pH elevation that facilitates metallic hydroxide precipitation and subsequent ion adsorption [62]. Zeta potential measurements indicate electrostatic metal adsorption predominates when solution pH surpasses biochar’s point of zero charge. Sludge-modified composite biochar enhances Cr adsorption through optimized physicochemical characteristics (Table 6) [63]. Increased pyrolysis temperatures and *P. crassipes* content structurally optimize biochar for improved adsorption [43,64,65]. *P. crassipes* biochar pyrolyzed at 393 °C shows peak lead adsorption capacity (195.24 mg/g) through combined electrostatic and pH-mediated mechanisms [62]. Iron/sludge composite biochars demonstrate specialized adsorption mechanisms including chemisorption and electron transfer, despite reduced capacities [43]. pH-dependent adsorption optimization varies metallospecifically, exemplified by the maximum removal of Cd at pH 9 [62]. These findings reinforce established pH-mediated adsorption paradigms [43,64,65].

**Table 6:** Comparison of Preparation Methods for *P. crassipes*-Derived Biochar.

Pyrolysis Feedstock	Pyrolysis Temperature (°C)	Adsorption Mechanism	Solution pH	Target Heavy Metal	Maximum Adsorption Capacity (mg/g)	Reference
*P. crassipes*, iron salts, K_2_CO_3_	300~500	Monolayer chemisorption	2.0	Cr	18.50	[39]
*P. crassipes*	393	Electrostatic attraction	7.0	Cu	177.66	[62]
			5.0	Pb	195.24	
			9.0	Cd	142.59	
			6.0	Zn	146.14	
*P. crassipes*, sludge	300~500	Electron donor–acceptor interaction	----	Cr	44.96	[63]

Note: Reference [63] does not provide detailed numerical descriptions of solution pH.

### 4.2. Limitations of P. Crassipes in Remediation of Heavy Metal-Contaminated Water

#### 4.2.1. Ecological Risks of *P. crassipes*

*P. crassipes* demonstrates exceptional ecological plasticity through broad environmental tolerance, establishing itself as a paradigm of aquatic invasiveness. Laboratory studies demonstrate ten initial specimens proliferating exponentially to 1610 individuals over ten-month growth cycles under optimal conditions [20]. Invasion ecology operates through tripartite environmental stress induction, where floating mat formation physically alters aquatic habitats by inhibiting gas exchange and attenuating light penetration, which concurrently depletes dissolved oxygen and suppresses photosynthesis in the photic zone, while hydrological modifications arising from biomass densities exceeding 60 kg/m^2^ wet weight amplify channel roughness indices, impair flow dynamics, and compromise hydraulic infrastructure functionality, because allelochemical exudations of phenolic compounds induce biochemical exclusion through the suppression of native species and the depletion of biodiversity, collectively driving ecosystem destabilization via synergistic mechanochemical interactions across coupled physical-hydrological-chemical pathways [56]. Invasion-mediated ecosystem cascades exhibit polymodal risk architectures [66].

Biomass accumulation rates positively correlate with fluvial obstruction probabilities [67]. Favorable conditions accelerate surface colonization, where hyperdense biomass induces hydrological obstructions, disrupts navigation logistics, and exacerbates flood-related infrastructure vulnerabilities. Supra-threshold coverage reduces thermal regimes, acid-base balance, and oxygen saturation, compromising natural bioremediation potential [64]. Monoculture establishment through competitive exclusion disrupts ecological stoichiometry [65]. Benthic biomass decomposition releases eutrophication accelerants while generating dipteran breeding microhabitats, collectively degrading habitat integrity [68]. Stress-responsive sexual reproduction yields persistent seed banks with secondary invasion latencies, complementing dominant vegetative propagation. The plant’s metallophyte characteristics introduce trophic transfer risks, as bioaccumulated heavy metals may biomagnify through agricultural reuse pathways [66].

#### 4.2.2. Secondary Pollution Risk of *P. crassipes*

The secondary pollution risks of *P. crassipes* primarily arise from technical shortcomings in control methods and environmental mismanagement during resource utilization, as these risks manifest through three specific mechanisms: chemical control poses ecological risks due to herbicide residues affecting non-target species, physical and biological control methods are constrained by incomplete biomass removal and potential reintroduction of invasive species, and ecological restoration projects face secondary contamination issues when improperly disposed biomass releases heavy metals [69].

Herbicide applications for *P. crassipes* management induce ecological risks through composite pollution. Although herbicides provide broad-spectrum effectiveness and operational efficiency, their large-scale use generates three principal adverse effects. Spatial variability in herbicide distribution frequently causes incomplete plant inactivation, suppressing surface biomass while failing to control submerged seed bank regeneration [70]. Prolonged herbicide exposure drives resistance evolution in *P. crassipes* populations, reducing the effectiveness of long-term control [70]. Additionally, residual herbicides bioaccumulate in aquatic organisms, causing sublethal impacts that disrupt trophic networks and potentially trigger irreversible ecological transitions through community restructuring [71].

Physical and biological control approaches present technical limitations. Manual harvesting achieves temporary biomass reduction but risks terrestrial heavy metal contamination through improper leachate management. Biological control using herbivorous species faces ecological uncertainties, including invasion risks and unpredictable species interactions. Documented cases demonstrate that biocontrol agents may exceed intended ecological ranges and alter the feeding patterns of non-target species [72].

The resource utilization of harvested biomass introduces environmental hazards through cross-media contamination. Incomplete pyrolysis during biofuel production generates persistent pollutants such as polycyclic aromatic hydrocarbons. Fertilizer production through fermentation increases heavy metal bioavailability, elevating soil contamination risks. Unprocessed biomass used as livestock feed enables toxin biomagnification across food chains, endangering animal and human health. The hyperaccumulation capacity of *P. crassipes* particularly intensifies pollutant re-release risks during material processing [73].

Effective environmental management requires the implementation of ecological engineering principles [74]. This involves integrated strategies combining real-time monitoring of population dynamics and pollutant concentrations, developing closed-loop resource utilization systems with strict process controls [75], and promoting interdisciplinary research linking invasion biology with material cycling mechanisms to optimize remediation approaches [76].

## 5. Conclusions and Future Directions

Looking ahead, this field of research must prioritize ecological adaptability research to enhance technology transfer led by China and India, address research gaps in Africa and South America, and overcome bottlenecks in technological localization through cross-regional collaboration. Concurrently, establishing a standardized management system that balances ecological safety and technology dissemination is critical to tackle challenges such as disparities in data quality and the lack of international standards.

*P. crassipes* demonstrates elevated biomass productivity and robust hyperaccumulator characteristics, achieving efficient heavy metal remediation and water purification via integrated physicochemical adsorption mechanisms. The extensive root architecture, substantial specific surface area, and abundant functional groups including carboxyl and hydroxyl groups enable effective metal sequestration. Biochar derivatives from this species exhibit augmented adsorption capacities, indicating scalable remediation potential. Comparative analyses reveal significantly enhanced remediation efficiency compared to alternative aquatic macrophytes, demonstrating broad-spectrum metal removal capabilities and operational stability under heterogeneous contamination conditions. *P. crassipes* exhibits notable resource recovery potential through applications in bioenergy production, agricultural fertilization, and animal feed formulations, generating dual ecological and socioeconomic benefits. Strategic development should prioritize the establishment of circular resource recovery systems while controlling secondary risks including the propensity for biological invasion and contaminant remobilization, thereby ensuring sustainable implementation within remediation frameworks. A tripartite regulatory mechanism comprising competitive adsorption modulation, intermetallic synergy potentiation, and rhizospheric sequestration optimization enables effective resolution of the inherent challenges in multiplexed metal remediation, particularly regarding simultaneous multi-target extraction and ionic interference suppression. Operational optimization requires systematic evaluation of intermetal interactions and physiological tolerance thresholds during deployment in polymetallic contamination systems. These adaptive mechanisms collectively establish *P. crassipes* as a robust phytoremediation candidate for complex aquatic metal pollution.

The ecological implications of *P. crassipes* colonization demand critical evaluation. This facultative macrophyte jeopardizes aquatic biodiversity through competitive exclusion of indigenous vegetation and structural habitat modifications, fundamentally destabilizing ecosystem equilibrium. Knowledge gaps persist regarding its context-dependent ecological plasticity and interspecific interaction dynamics, constraining its strategic deployment in restoration initiatives. Ecological management complexities arise from dual interconnected factors. Exponential biomass expansion accelerates eutrophication, compromises hydrological integrity, and induces biotic homogenization via the establishment of monocultural dominance. Conventional containment strategies involving chemical applications, mechanical harvesting, or biological controls demonstrate inherent technical constraints including evolved herbicide tolerance, residual biomass retention, and variable biocontrol performance, potentially exacerbating ancillary pollution pathways. Resource valorization processes carry inherent risks of heavy metal remobilization and suboptimal pyrolysis byproduct management. Mitigating these multidimensional risks necessitates implementing rigorous ecological risk evaluations coupled with holistic management frameworks that reconcile remediation efficacy with ecosystem conservation imperatives.

Future studies should prioritize optimizing *P. crassipes* growth conditions by enhancing heavy metal adsorption capacities via CRISPR-based genetic modifications and environmental parameter adjustments, such as pH regulation and nutrient supplementation. Concurrently, investigations into ecological adaptability under variable temperature and salinity gradients, alongside synergistic interactions with co-occurring aquatic species like P. stratiotes and Lemna minor, are essential for designing multi-species remediation systems. To mitigate ecological risks, invasion management strategies integrating biocontrol thresholds and real-time monitoring must be developed, complemented by evaluations of secondary pollution pathways during field-scale deployments. Innovations in resource utilization should focus on biochar composites and nanocellulose extraction to improve economic viability, supported by standardized protocols for toxin screening in biomass conversion and pyrolysis optimization for biofuel production. Transnational data-sharing platforms and technical standardization frameworks will enhance geographical specificity and global scalability of these technologies. Furthermore, elucidating rhizosphere microbiome interactions will enable engineering of phytoremediation-microbial consortia with improved efficiency and stability. Addressing these priorities will facilitate sustainable integration of *P. crassipes* into circular economies while maximizing ecological and socioeconomic benefits.

## Figures and Tables

**Table 1:** The toxic effects of different heavy metals on various organisms/environments [16,17].

Heavy Metal	Ionic Form/Complex Compound	Species/Subject	Toxicity Specific	Heavy Metal Treatment Conditions
Cr^6+^	Cr_2_O_7_^2−^ (from K_2_Cr_2_O_7_), CrO_4_^2−^, Phosphate complex	*Vibrio fischeri*	EC_50_: 2.65 mg/L	30 min, luminescence inhibition
		Activated sludge	EC_50_: 5.84 mg/L	30 min, respiration inhibition
		*Oryzias latipes*	LC_50_: 2.30 mg/L	96 h exposure, lethality test
		*Daphnia magna*	EC_50_: 3.20 mg/L	24 h exposure, reproduction inhibition
		*Pseudokirchneriella subcapitata*	EC_50_: 0.30 mg/L	72 h exposure, growth inhibition
Zn^2+^	Zn^2+^ (from ZnSO_4_⋅7H_2_O), Bovine serum albumin complex	*Vibrio fischeri*	EC_50_: 22.74 mg/L	30 min, luminescence inhibition
			EC_50_: 20.93 mg/L	60 min, luminescence inhibition
		Activated sludge	EC_50_: 30.0 mg/L	24 h exposure, enzyme activity inhibition
		*Brachydanio rerio*	LC_50_: 2.50 mg/L	96 h exposure, lethality test
		*Daphnia magna*	EC_50_: 1.00 mg/L	24 h exposure, survival inhibition
		*Desmodesmus subspicatus*	EC_50_: 0.50 mg/L	72 h exposure, growth inhibition
Cu^2+^	Cu^2+^, Complex with metal sites at dehydrogenase active center	*Pseudomonas putida*	EC_50_: 21.4 mg/L	16 h, growth inhibition
		Activated sludge	EC_50_: 0.50 mg/L	24 h exposure, enzyme activity inhibition
		*Crassius auratus*	LC_50_: 0.30 mg/L	24 h exposure, lethality test
		*Daphnia magna*	EC_50_: 0.05 mg/L	24 h exposure, reproduction inhibition
		*Pseudokirchneriella subcapitata*	EC_50_: 0.03 mg/L	72 h exposure, growth inhibition
Cd^2+^	Cd^2+^, Complex with sludge organic matter	Activated sludge	EC_50_: 5.0 mg/L	30 min, respiration inhibition
		*Vibrio fischeri*	LC_50_: 10.0 mg/L	30 min, luminescence inhibition
		*Oryzias latipes*	EC_50_: 0.50 mg/L	96 h exposure, lethality test
		*Daphnia magna*	EC_50_: 0.10 mg/L	24 h exposure, survival inhibition
		*Desmodesmus subspicatus*	EC_50_: 0.08 mg/L	72 h exposure, growth inhibition
Ni^2+^	Ni^2+^, Phosphate complex in medium	Activated sludge	EC_50_: 2.0 mg/L	24 h exposure, enzyme activity inhibition
		*Vibrio fischeri*	EC_50_: 15.0 mg/L	30 min, luminescence inhibition
		*Brachydanio rerio*	LC_50_: 3.0 mg/L	96 h exposure, lethality test
		*Daphnia magna*	EC_50_: 1.0 mg/L	24 h exposure, reproduction inhibition
		*Pseudokirchneriella subcapitata*	EC_50_: 0.20 mg/L	72 h exposure, growth inhibition

**Table 2:** Top five countries and institutions by publication volume.

Ranking	Country and Institution	Total Number of Publications	Total Citation Count	Average Citations per Paper
C1	India	100	2941	29.41
C2	China	68	2071	30.46
C3	Egypt	23	792	34.43
C4	Pakistan	19	545	26.45
C5	Saudi Arabia	19	507	26.31
I1	King Khalid University(Saudi Arabia)	6	133	22.17
I2	National Institute of Oceanography and Fisheries (Egypt)	6	113	18.83
I3	King Saud University (Saudi Arabia)	5	55	11.00
I4	Tanta University (Egypt)	4	152	38.00
I5	Government College University (Pakistan)	4	100	25.00

Note: C1, C2, C3, C4, and C5 represent the top 5 countries in the rankings. I1, I2, I3, I4, and I5 represent the top 5 research institutions in the rankings. C, country; I, institution. Dataset date range: 1 January 1980 to 7 May 2025.

**Table 3:** Purification efficiency of *P. crassipes* on Cu^2+^ ions.

Species	Target Site	Purification Mechanism	Cu Solution pH	Peak Purification Efficiency (%)	Reference
*P. crassipes*	Root	Ion exchange and complexation	4.0–6.5	75%	[23]
*P. crassipes*	Stem	Electrostatic attraction	4.5	97%	[26]
Long-root *P. crassipes*	Whole plant	Electron donor–acceptor interactions	6.0	----	[28]
*P. crassipes*	Root	Ion exchange	4.0–6.5	75%	[29]

Note: The parameter of peak purification efficiency is derived from the data in [23,26,28,29] but these studies did not provide detailed numerical descriptions of peak purification efficiency.

**Table 4:** Purification efficiency of *P. crassipes* for chromium solutions at different concentrations.

Chromium Concentration in Solution (mg/L)	Root Removal Rate (%)	Stem Removal Rate (%)	Leaf Removal Rate (%)
2	65.0%	27.4%	16.4%
4	56.0%	---	---
6	57.0%	---	---
8	58.0%	34.0%	11.0%

Note: The purification efficiency values for Cr^6+^ by *P. crassipes* are based on peak purification values cited from the literature [37]. The ‘---’ indicates data not obtained from the experiment. According to [37], the purification time of *P. crassipes* for Cr^6+^ is 11 days.

**Table 5:** Purification efficiency of chromium ions by *P. crassipes*.

Material	Purification Mechanism	Peak Efficiency (%)	Cr Solution pH	Adsorption Type	Reference
Root	Biosorption and phytostabilization	55%	----	Physical adsorption	[37]
Root	Root accumulation	84%	----	Physicochemical synergistic adsorption	[38]
Whole plant	Redox reaction, electrostatic attraction, and complexation	85.7%	2.0	Physicochemical synergistic adsorption	[39]

Note: Reference [37] does not specify the PH of the Cr solution, reporting a 15-day purification period for Cr^6+^ (as K_2_Cr_2_O_7_) by *P. crassipes*. Reference [38] documents an 11 day purification cycle. In practice, magnetic *P. crassipes* biochar (MBC) is used for Cr^6+^ wastewater purification, with the plant serving solely as the raw material. The adsorption equilibrium time of MBC for Cr^6+^ is 6 h [39].

## Data Availability

All data generated or analyzed during this study are included in this published article.
